# Disgust Processing and Potential Relationships with Behaviors in Autism

**DOI:** 10.1007/s11920-023-01445-5

**Published:** 2023-09-06

**Authors:** Aditya Jayashankar, Lisa Aziz-Zadeh

**Affiliations:** 1 USC Mrs. T.H. Chan Division of Occupational Science and Occupational Therapy, University of Southern California, Los Angeles, CA 90089 USA; 2 Brain and Creativity Institute, Dornsife College of Letters, Arts and Sciences, University of Southern California, Los Angeles, CA 90089 USA

**Keywords:** Disgust, Autism, Somatic marker, Moral disgust

## Abstract

**Purpose of Review:**

While there are reports of differences in emotion processing in autism, it is less understood whether the emotion of disgust, in particular, plays a significant role in these effects. Here, we review literature on potential disgust processing differences in autism and its possible associations with autistic traits.

**Recent Findings:**

In autism, there is evidence for differences in physical disgust processing, pica behaviors, attention away from other’s disgust facial expressions, and differences in neural activity related to disgust processing. In typically developing individuals, disgust processing is related to moral processing, but modulated by individual differences in interoception and alexithymia.

**Summary:**

Autistic individuals may experience atypical disgust, which may lead to difficulty avoiding contaminants and affect socio-emotional processing. In autism, such outcomes may lead to increased occurrences of illness, contribute to gastrointestinal issues, diminish vicarious learning of disgust expression and behaviors, and potentially contribute to differences in processes related to moral reasoning, though further research is needed.

**Supplementary Information:**

The online version contains supplementary material available at 10.1007/s11920-023-01445-5.

## Introduction

It is well known that autistic children face differences in emotional processing; however, it is less understood how some of these differences may be specifically associated with the emotion of disgust. Disgust is a negative basic emotion, mostly associated with “bad taste” [1–3, 4•, 5, 6]. Basic emotions (disgust, happy, sad, anger, surprise, fear) are a special set of emotions that are the most distinct and universal across cultures and species and most related to survival [5]. Disgust is thought to be one of the earliest emotions to have been developed evolutionarily due to how critical it is for avoiding contaminants and increasing survival [7–9]. Furthermore, feelings of disgust may have evolved to serve as the basis of feelings of contempt and socio-moral evaluations [10]. Autistic children may show differences in disgust processing, potentially leading to pica behaviors, gastrointestinal (GI) disturbances, and socio-emotional issues [11, 12]. However, despite its importance, disgust has been understudied and often rolled together with research on other basic emotions or research on sensory sensitivities. In this narrative review, we introduce the topic of disgust and then explore data on potential disgust processing differences in the autistic population and how they may impact other symptomatology.

## Disgust: Background

Most present-day definitions of disgust are derivations of Angyal’s [13] description as *the repulsive response to the thought of orally ingesting some aversive or offensive material*. Disgust is thought to have evolved in humans as an embodied defense mechanism against contamination and, by extension, society from potentially toxic substances or situations [14, 15]. Damasio [10] has described the social dimension of disgust as akin to contempt. He characterizes contempt as a “biological metaphor for disgust” and acts as a somatic marker that elicits a rejection response to immoral or toxic behaviors, similar to how physical disgust protects us from toxic substances (*Somatic Marker Hypothesis*: [10, 16–18]). This feeling of contempt would have informed early civilizations about the morality of actions, thus laying the foundation for ancient forms of law and socio-cultural norms [19].

Elicitors of disgust may be categorized into different domains. Rozin and colleagues [20] suggested a four-factor model to explain different domains of disgust elicitors: core (i.e., disgusting foods, smells, objects), animal reminder (e.g., injections), interpersonal (e.g., unwanted contact with strangers), and socio-moral disgust (e.g., violating a social norm). Alternatively, Tybur and colleagues [21] proposed a three-factor functional model that includes: pathogen-related disgust (which combines Rozin’s core, animal reminder, and interpersonal contact domains), sexual disgust, and socio-moral disgust. Examples of these situations and descriptions of the domain-specific behavioral signatures can be found in Table 1. In this review, in keeping with relevance to autistic young individuals (henceforth used to refer to both children and adolescents), we focus primarily on core pathogen-related and socio-moral disgust. The methodology for article search can be found in the Supplemental Materials.

**Table 1:** Description of the different domains of disgust as per the Rozin et al. and Tybur et al. models [20, 21]

Disgust domain (Rozin)	Disgust domain (Tybur)	Behavioral signature	Situation examples
*Core/physical*	*Pathogen-related*	• Stimuli that traditionally elicit physical revulsion • Stimuli or acts elicit disgust because of their potentially contaminating or nauseating nature	• Feces • Vomit • Rotten foods
*Animal reminder*		• Elicitors *MUST* be common between humans and animals • Felt disgust is rooted in feelings of how similar the animal “act” is to oneself or humans, in general • Overlaps with core disgust elicitors, but differs in the nature of felt disgust	• Defecation • Childbirth • Body envelope violations, like mutilation or injections • Sexual acts (*separate domain in Tybur model*)
*Interpersonal*		• Includes stimuli that pose a threat to one’s own personal space • Disgust elicits feelings of strangeness, the threat of contamination	• Interaction with strangers • Interaction with amputees
*Socio-moral*		• Includes stimuli that pose a threat to one’s own personal values or conscience • Disgust elicits feelings of immorality/taboo or impurity • Caused by uniquely human acts and social cues	• A maskless individual during a pandemic • Public urination • Incestuous relationships

## Disgust in Autism

### Core Disgust in Autism: Behavior

The literature on disgust experiences in autism is considerably limited [22•], due to researchers commonly studying disgust alongside other basic emotions and general emotion and sensory processing differences [23, 24••, 25•]. About 53–94% of autistic individuals experience sensory sensitivities [26], some of which may contribute to the feeling of disgust. There is evidence for both smell hyposensitivity [27•, 28–30] and hypersensitivity [31] in autism, which may vary as a factor of age. Furthermore, smell detection distances have been correlated with autistic symptomatology [31]. Olfactory and gustatory sensitivities in particular may play a critical role in the development of food behaviors in autistic children [32, 33]. Particularly, the appraisal of food odors as pleasurable or disgusting and individual olfactory sensitivity was found to significantly influence reluctance to eat new foods in autistic children, but not in the comparison group [33, 34]. However, it has not been established if sensory sensitivities in olfaction or gustation are necessarily linked to higher disgust feelings. It is possible that they are simply more unpleasant rather than specifically invoking disgust. Given that there are many food selectivity issues in autism [35], further studies are needed to better understand how disgust processing may serve as a mediating factor.

Autism is a highly heterogeneous condition and may encompass a wide range of disgust proneness traits that affect food-related behaviors [36, 37]. Disgust proneness [38–41] encompasses three aspects that moderate individual differences in the experience of disgust: *disgust propensity* (how often you feel disgusted; [42]), *disgust reactivity* (how disgusted you are by an aversive stimulus; [41]), and *disgust sensitivity* (how bad you feel after feeling disgusted; [41, 43]). Previous research has reported that about 50% of youth with ASD exhibit unusual patterns of physical disgust sensitivity [24••]. For instance, potentially indicating lower disgust proneness, young autistic individuals are more likely to eat items that were not meant for consumption, with about 23% displaying pica behaviors [44]. On the other hand, in autism, higher levels of sensory sensitivities are thought to be the main influence on food selectivity, and such sensitivities may interact with disgust proneness [37]. Such interaction may present itself as heightened disgust proneness [45], resulting in more food pickiness. One of the ways children learn about food and contamination-related behaviors during development is through social learning and communication [46–48], both of which may be considerably challenging for young autistic individuals [49, 50] and may underlie their diminished contamination sensitivity [24••, 51]. A reduced sensitivity to processing others’ negative emotions may further cause difficulties with disgust learning in autistic individuals [52, 53].

There is also increasing evidence that about 9–91% of autistic children suffer from GI symptoms [54–56]. Given that disgust processing disturbances may affect food preferences (i.e., eating foods not meant for consumption or, more subtly, preference for high-carbohydrate foods), issues in disgust processing may indirectly exacerbate GI problems in autism [57–59, 60•]. In general, few studies focus on potential differences in core disgust processing in autistic individuals and further research on this topic is needed [11, 24••, 51].

### Core Disgust in Autism: Neurobiology

Are there neurological signatures of potential differences in disgust processing in autism? To answer this question, we first discuss the general neural substrates of disgust processing.

Early stimulation studies revealed the insula as a hub for processing nauseated behaviors [61]. Lesion studies found that while several limbic structures were involved in disgust processing [62, 63], damage to the insula in particular was associated with difficulty recognizing disgust, indiscriminate food consumption, and the absence of contaminant-avoiding behavior [64]. Thus, while the insula and associated regions are important in the processing of many emotions, there is evidence that the anterior insula (AI) in particular seems critical for processing core disgust [65]. Furthermore, the AI has also been implicated in processing disgusted facial expressions [66–68] and may be an important neural correlate of moral disgust processing [69, 70]. Neuroimaging evidence suggests that dysfunctional integrity and connectivity within the AI can result in divergent disgust experiences, for both core and vicarious disgust [71]. Furthermore, individual differences in disgust proneness, socio-cultural factors, and educational environments may modulate neural activity associated with disgust [22•, 72–74].

In autistic individuals, the differential functional integrity of the AI compared to peers has been consistently found to be associated with difficulties with emotion, empathic, and social processing, including disgust-related processing [75–79, 80••, 81, 82]. Hence, atypical functional activity and connectivity in the AI in young autistic individuals may underlie difficulties with disgust emotion processing. However, the insula is a complicated structure, working alongside networks of regions [83, 84], and involved in a multitude of social [85, 86], emotional [87, 88, 89•, 90], sensory [91] (see also *Somatic Marker Hypothesis*: [16–18, 92]), and cognitive tasks [93, 94]. Indeed, disgust may activate a network of regions between the insula and frontotemporal regions involved in interoceptive, emotional, and socio-cognitive processing [95]. It has been argued that the AI, in part, processes domain-specific disgust experiences and serves as a hub for disgust experiences, managing inputs from emotion-related regions (amygdala, striatum, putamen, medial prefrontal, orbitofrontal, sensory, and anterior cingulate cortices) as well as from the viscera [22•]. How these networks uniquely differ during core disgust processing in autism remains to be better understood [96].

### Facial Recognition and Vicarious Learning of Disgust in Autism

Around the age of seven, children learn about disgust vicariously, by watching others or hearing of others having disgust experiences [97]. The ability to recognize another person’s disgusted facial expression, a first step to vicarious disgust processing, involves neural activity in facial emotion recognition (FER) circuits (inferior occipital regions, fusiform face area [FFA], the posterior superior temporal sulcus [pSTS]), and emotion-related brain regions like the insula and amygdala [39, 97].

Interestingly, personally feeling disgusted and observing another person experiencing disgust tend to activate the same regions in the AI and anterior cingulate cortex (ACC) [98, 99], suggesting shared circuits for self and vicarious disgust processing [99–101]. Vicario and colleagues [74] further found that individual differences in disgust sensitivity predicted not only the suppression of tongue motor activity (cortico-hypoglossal inhibition) upon viewing disgusting stimuli, but also upon viewing others experiencing disgust. Taken together, these results indicate that we implicitly mirror others’ experiences of disgust [74].

In autistic individuals, many studies have found compelling evidence of difficulties with FER, especially for others’ disgust and anger facial expressions [23, 25•, 102–105, 106••, 107••]. Behaviorally, for example, Zhao and colleagues [25•] found that young autistic individuals exhibit increased attention bias and hypervigilance when viewing disgusted facial expressions, which leads to avoidant gaze behavior toward the entire set of emotional face stimuli. Similarly, Yeung and colleagues [106••] found that young autistic individuals may have emotion-specific FER difficulties (i.e., for specific negative emotions, like disgust) above and beyond differences in face processing seen in autism. Thus, differences in processing disgust faces may impact other socio-emotional processing. These differences may have important downstream implications for the learning of disgust stimuli and appropriate disgust responses from parents, peers, and teachers.

Neuronally, a number of brain regions including the ACC, precuneus, fusiform, inferior frontal gyrus pars opercularis (IFGop), and amygdala may be differentially activated in FER in autism [86, 102, 108–117] and differences may be impacted by development [118]. However, most studies have focused on numerous facial expressions, not just disgust facial expressions, making it difficult to infer disgust-specific neuronal FER differences in ASD. One study by Bastiaansen and colleagues [118] explored potential differences between viewing disgust, pleasure, and neutral facial expressions in autistic versus non-autistic participants and found no group by emotion differences. However, looking across facial expressions, they found that younger individuals in the autistic group showed lower activity in the IFGop compared to both a typically developing group and a group with schizophrenia [118], and that activity in the IFGop correlated with autistic symptomatology, a finding that has also been shown in other studies that considered observation of facial expressions of different emotions in autistic populations [108, 110]. To better understand if disgust facial expressions may be neuronally processed differently than other negative emotions in autism, further studies are necessary.

### Socio-moral Disgust in Autism

#### Background

Rationalist theories of morality dominated early thinking of moral development and decision-making [119–122]. These theories purported the importance of understanding the intentions of the moral agents via the theory of mind (ToM) or mentalizing, role-taking, recognition of the victim’s emotional state, and empathic processing. However, more recently, another moral decision-making theory, the “social intuitionist theory” [123] has developed to suggest a critical interplay between disgust processing and moral processing, as well as the impact of culture in influencing moral reasoning [20, 124, 125]. The intuitionist theory is in many ways similar to Damasio’s theory on feelings of contempt arising from disgust [10]. It suggests that in the same way, physical disgust deems smelly food “bad,” negative evaluations of social norm violations can lead to feelings of “impurity” which influence aversive behaviors directed towards immoral agents, deemed as “bad and harmful” [126–128]. Moral foundations theory [129–131], an extension of social intuitionist theory, redefines these moral intuitions into moral foundations (care/harm, fairness/cheating, loyalty/betrayal, authority/subversion, sanctity/degradation, liberty/oppression), developed to resolve social problems [130, 132, 133]. Of these moral foundations, sanctity (or purity) violations (degrading acts [i.e., drunken groping], sexually deviant acts, or acts that pose a risk of contamination [i.e., urinating in a public pool]) have been most closely linked with the emotion of disgust [134••, 135, 136]. Supporting this claim, sanctity violations have been associated with disgust facial responses [137, 138]. However, some researchers suggest that disgust is likely linked with all the moral foundations to some degree [135, 139–142].

In support of these theories, in non-autistic participants, prior work indicates that the perception of a physically disgusting stimulus can bias moral judgment during evaluations of moral actors [99, 141, 143••, 144]. This may be due to core and socio-moral disgust both inducing the same oral-nasal rejection behavioral and facial responses [69, 138, 145]. Cannon and colleagues [137] found the disgust facial response was most associated with moral transgressions involving violations of purity and fairness, while anger facial responses were more associated with harm violations. Additionally, they found that the intensity of the facial response corresponded with the perceived severity of the moral violations [137], with similar findings in children [146].

Furthermore, feelings of core and socio-moral disgust may be manipulated using olfactory stimuli that either inhibit or elicit nausea [99, 140, 141, 147]. Wheatley and Haidt [142] found that the sensation of an extrinsically disgusting odor can elicit more stringent moral evaluations. Expanding on this relationship, Schnall and colleagues [140] conducted a series of experiments to test the effect of extrinsic disgusting stimuli on moral evaluations. They found that the perception of a disgusting odor, being in a disgusting room, watching disgusting videos, or vividly remembering a disgusting experience can elicit more stringent moral evaluations. However, in some cases, these results only held true in participants with higher levels of interoceptive awareness [140].

Disgusting smells can also influence feelings of interpersonal trust, which may undermine social trust and relationships and bias moral evaluations of others. For instance, Lee and colleagues [148] found priming participants with a fishy odor (an embodied metaphor of suspicion) induced higher feelings of mistrust. Finally, one study found that the inability to differentiate emotions, a common feature of alexithymia, was found to predict reduced effects of disgust priming on moral judgments [149]. Taken together, there is evidence to support intuitionist/moral foundation theory, with studies indicating a strong relationship between physical and moral disgust processing, though these may be modulated by interoceptive ability and the presence of alexithymia. Thus, in autism, where there are common co-existing differences in disgust processing [24••], interoceptive awareness [150], and/or high co-occurrence of alexithymia [151, 152], one may expect differences in moral judgments. These, in turn, could adversely affect social relationships [153], if not properly understood.

#### Socio-moral Processing in Autism

Most studies on autism have focused on mentalizing abilities, in line with rationalist theories of moral judgments [154–160]. These studies indicate that young autistic individuals show differences in mentalizing ability when compared to non-autistic peers, though they can often ascribe others’ mental states just as well as non-autistics with more time [154, 160]. In addition, one review indicated that in judging accidental situations (i.e., accidentally causing someone to fall while playing and running around), autistic individuals tend to have more outcome-based reasoning compared to non-autistic peers, who instead relied more on intent-based reasoning [161]. Another study in autistic adults also found differences in moral reasoning for transgressions of others during a social intentionality task (e.g., a confidant giving away embarrassing information either purposely or by mistake), in which autistic individuals made more rigid and relatively more stringent judgments for both intentional and unintentional actions as compared to non-autistic individuals [162]. Understanding these potential differences in reasoning is important, because stricter and more stringent judgments by some autistic children may result in awkward social interactions with non-autistics, loss of friendships or acquaintances, and social ostracism [163].

On a closer look, while several studies indicate autistic individuals tend to prioritize outcomes over intents in justifying moral decisions [164••], some researchers found that autistic children tend to make similar judgments as peers without autism [134••]. Incidentally, punishment recommendations in the autism group were more predicted by perceived wrongness of transgressions rather than autistic traits [134••]. Nevertheless, autistic children were found to make significantly more punishment recommendations for moral transgressions than the comparison group (particularly for social norm violations [134••]). By contrast, Margoni and colleagues [165•] found that autistic children were able to make intent-based moral judgments and that previous differences could be explained by the prevalence of executive dysfunction and difficulties inhibiting a prepotent outcome response [151, 162, 166]. Furthermore, autistic individuals may require that the moral agent’s intentions be explicitly described in order to make intent-based moral judgments [162, 167], while unclear or vague descriptions of intentions will predispose them to making outcome-based moral decisions and more stringently meting out punishment to unintentional acts by moral agents [168–170]. This is consistent with prior reports of differences in intention understanding in autism [161, 169, 171]. Interestingly, one study found that autistic and alexithymic traits had opposite effects on utilitarian decision-making during a moral decision-making task (the trolley problem), with higher alexithymic traits (rather than autistic traits) resulting in more utilitarianism [158]. Taken together, prior research indicates that there may be an interplay between intention understanding, executive functioning ability, and alexithymia in moral reasoning in autism.

How might disgust processing differences also impact differences of moral reasoning in autism? Is there also evidence for the intuitionist/moral foundation theory in autism, with disgust impacting the processing of moral reasoning? Previously, we discussed the heterogeneity within autism, which could possibly lead to a range of disgust proneness [36, 37], interoception ability, and levels of alexithymia. However, given some autistic individuals experience potential differences in disgust processing [24••], interoception ability [150, 172–174], and increased prevalence of co-occurring alexithymia (55%; see [175, 176•]), all of which are known to impact moral decisions in non-autistic populations, one may expect a dynamic interplay of these factors in some autistic children [150, 177]. However, to our knowledge, there have not been extensive studies on this topic, and further work is needed to better understand the potential impact of disgust processing differences (alongside alexithymia and interoception processing differences) with potential differences in moral reasoning in autism. Given that such outcome-oriented decisions may dampen cooperative social behavior in autistic children, leading to social ostracism [163], a better understanding of such factors could facilitate improved social relationships for autistic children, all of which we discuss in the sections below.

#### The Potential Importance of an Intuitionist and Neurodiversity Approach

According to Haidt’s intuitionist approach [123] and the philosophy of neurodiversity [178–181], autistic individuals may recruit more learned rules-based resources and fewer emotion-based resources, and, as a consequence, even if they do not show differences with moral decision-making, their sources for moral judgments and moral development may prioritize different foundational domains [164••]. For example, in autistic individuals, from an intuitionist perspective, respecting authority and following rules could be the driving force behind making more outcome-based moral, rule-bound decisions [161, 162, 182, 183]. This may interact with co-occurring differences in disgust and interoceptive processing in autism, as well as the increased incidence of alexithymia, all leading to differential moral reasoning; namely less emotion-based decisions and more rule-based decisions. Indeed, there is evidence that hunter-gatherer societies also engage in outcome-based (rather than intention-based) moral reasoning and that the role intentions play in moral reasoning may instead depend on culture and context (see *weak moral intent hypothesis*, [177]), consistent with the notion that discussing these differences as “deficiencies” may not be appropriate. This unique neurodiverse perspective offers an alternate approach toward understanding potential differences in the processing of moral attributions in autistic individuals, but requires more research.

**Fig. 1:**
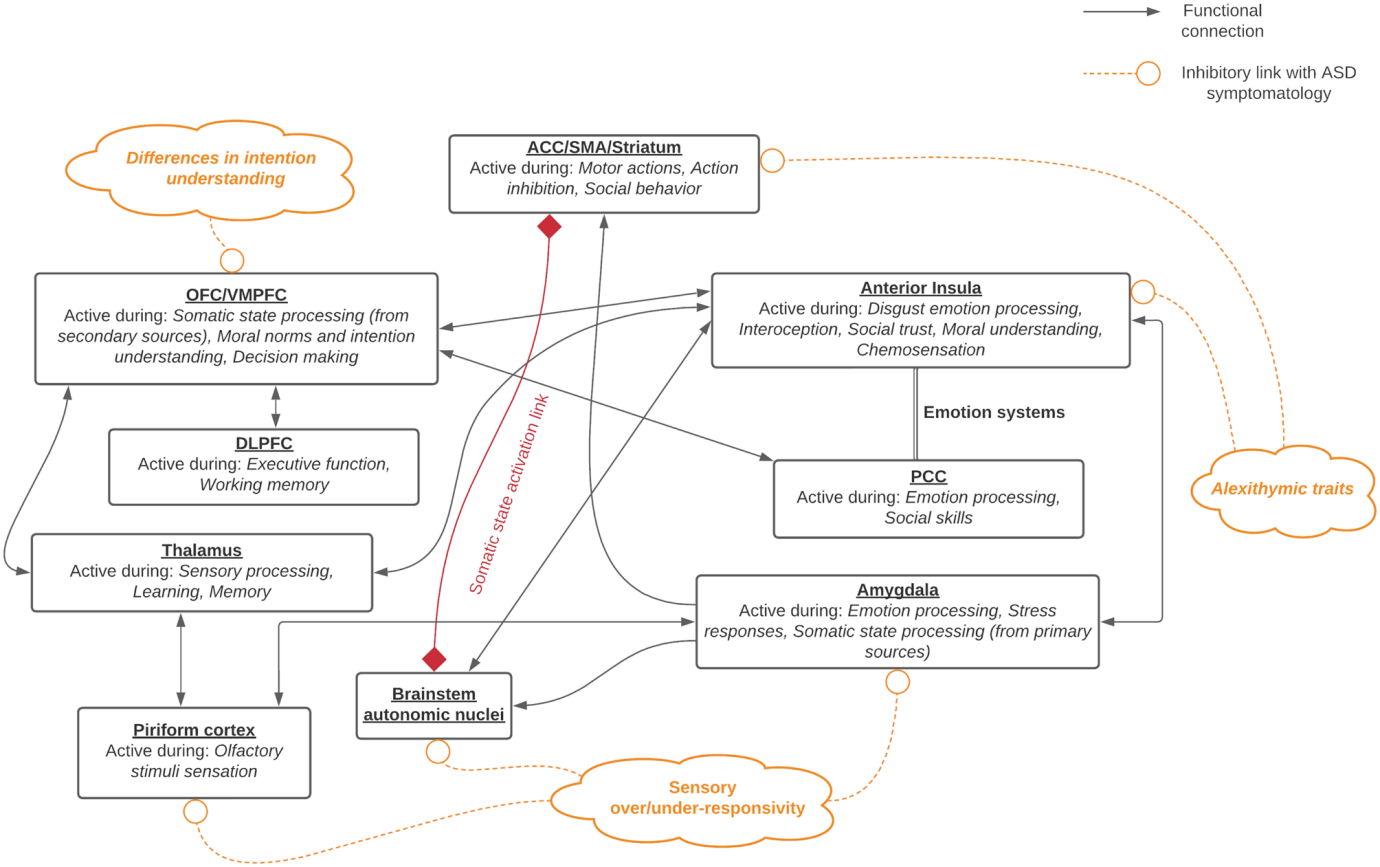
Conceptual model of the relationship between disgust, sensory, and moral processing regions in the brain and the hypothesized effect of autistic traits on these functional connections. Functional links are shown in arrows with triangular tips, somatic state linkage is shown with an arrow with diamond tips, and potential effects of autistic traits are shown in dashed arrows with rounded tips. ACC, anterior cingulate cortex; VMPFC, ventromedial prefrontal cortex; OFC, orbitofrontal cortex; DLPFC, dorsolateral prefrontal cortex; PCC, posterior cingulate cortex; SMA, supplementary motor area. Adapted from the Somatic Marker Hypothesis model in Bechara 2013; Koob et al. 2019; Saive et al. 2014 [193–195]

In contrast to the rationalist approach to understanding moral behaviors in autism — which has been the dominant way of investigating moral judgments in autism to date — an embodied cognition perspective could explore potential neuropsychological links between disgust processing and moral decision-making in young autistic individuals. This appears to be an open area of investigation, with startlingly few prior studies. Future studies in this area could help better understand how seemingly disparate autism symptomologies (sensory sensitivities, disgust processing differences, pica and food pickiness, potential interoception issues, alexithymia, FER disturbances, outcome-based moral decisions), may be interrelated and share common neural substrates and interactions. Such a theory could exist in parallel to those stressing the impact of executive functioning and ToM difficulties in autism, both providing avenues for interventions that can improve social relationships once better science is applied. We note that such theories for decision-making and mentalizing processing being influenced by emotion processing have been previously described in research related to the *Somatic Marker Hypothesis* [17, 18, 79, 184–188]. Thus, in Fig. 1, we adapt the somatic marker hypothesis model as a proposal of one possible conceptual neural network representation highlighting the relationships between the disgust, sensory, and moral processing neural systems.

## Conclusions

Here we summarized the literature on disgust processing and disgust-relevant behaviors in autism and compared it with research conducted on non-autistic individuals. There is evidence for behavioral and neurological differences in disgust processing in autism, in particular with physical disgust, as well as with processing other people’s disgust experiences, though further studies focusing particularly on disgust are necessary. In addition, in the future, this literature will need to be expanded to encompass individual differences on disgust propensity in autism, much like the sensory processing research has focused on both hypo- and hyper-individual sensory sensitivity differences. While we found no prior studies directly focusing on moral disgust in autism, based on the existing literature in non-autistics, we believe this is a rich avenue for future inquiry. It will be especially interesting to probe for potential relationships between alexithymia, interoception, disgust processing, sensory sensitivities, and moral disgust in autism both behaviorally and neurologically.

Indeed, further research on disgust processing in autism is important, due to the impact of disgust on health and activities of daily living. Young autistic individuals are prone to pica behaviors [44] and food selectivity [37], which can lead to further GI distress, disease, or toxicosis. Additionally, autistic children also have an early attentional bias away from attending to disgusted facial expression [25•, 189]. This bias away from attending to disgusted faces, especially those of one’s caregivers, may preclude the proper processing of disgusted faces required for vicarious learning of disgust [25•] during development. Thus, inefficient disgust processing and disgust learning in young autistic individuals may affect their physical, mental, and social health. Furthermore, given the tendency to make stricter judgments of unintentional actions, young autistic individuals may face difficulties with forming social relationships [162] and cooperative behavior [190], though data on alterations in disgust processing impacting these social judgments in autism remains to be determined. Furthermore, there is a need for a neural model in autism that better explains the potential impacts of sensory (exteroceptive and interoceptive) and emotional processing on cognitive, executive, and decision-making processes. How this model may interact with current Bayesian [191] and allostatic learning models of autism [192] remains an exciting future prospect.

### Supplementary Information

Below is the link to the electronic supplementary material.

Supplementary file1 (DOCX 15.3 KB)

## Data Availability

No datasets were generated or analysed during the current study.

## Declarations

### Disclaimer

Opinions, interpretations, conclusions, and recommendations are those of the authors and are not necessarily endorsed by the Department of Defense.
