# Supplementary material for: Disgust Processing and Potential Relationships with Behaviors in Autism
Source: Curr Psychiatry Rep. 2023 Sep 6;25(10):465–78. doi: 10.1007/s11920-023-01445-5 (PMC10627949; doi:10.1007/s11920-023-01445-5)
Supplement: Supplementary file 1 — Supplementary file1 (DOCX 15.3 KB) [file 11920_2023_1445_MOESM1_ESM.docx]

**Supplemental Materials: Search Strategy and Selection Criteria Methodology**

In this review, our aim was to identify similarities and/or differences in disgust processing, disgust-related behaviors, and neural circuitry in young autistics compared to peers without autism. Given the paucity of research on disgust in autism, we conducted a narrative review, instead of a scoping or systematic review, with the aim of broadly discussing this topic within the larger context of existing disgust research in general [196]. We searched Google Scholar, PubMed, PsycInfo, and ScienceDirect for peer-reviewed, English language articles and dissertations published between 1990 and 2022 with the use of an array of keywords, such as: [‘*disgust*’ OR ‘*disgust processing*’ OR ‘*disgust sensitivity*’ OR ‘*contamination sensitivity*’] AND [‘*autism*’ OR ‘*autism spectrum*’ OR ‘*autistic*’ OR ‘*ASD*’ OR ‘*ASC*’]. Additional search terms for specific domains of disgust included: [‘*core disgust*’ OR ‘*physical disgust*’ OR ‘*food-related disgust*’]; [‘*moral disgust*’ OR ‘*morality*’ OR ‘*moral decision*’]; [‘*social disgust*’ OR ‘*social processing of disgust*’ OR ‘*facial processing of disgust*’ OR ‘*vicarious disgust*’]. For PubMed, MeSH terms used included “*autism spectrum disorder*”, “*disgust*”, and “*morals*” (when searching for articles on moral disgust only). Additional studies were identified through the references of articles found. Every article was critically reviewed and included in the review it satisfied a set of criteria. Articles were deemed appropriate based on relevant research on the autism spectrum, reported distinct data on the disgust emotion processing or moral processing in autism, validity of methods used to study disgust processing, reliance on well-established measures of disgust, and clarity of the reported findings. Additionally, we adapted recent suggestions for avoiding ableist language into the language of this review [197].
